# Better than expected? Predictors of coping with expectation violations in the communication about death and dying

**DOI:** 10.3389/fpsyg.2023.1256202

**Published:** 2023-10-30

**Authors:** Yannik Bendel, Chrys Gesualdo, Martin Pinquart, Pia von Blanckenburg

**Affiliations:** ^1^Clinical Psychology and Psychotherapy, Department of Psychology, Philipps University of Marburg, Marburg, Germany; ^2^Developmental Psychology, Department of Psychology, Philipps University of Marburg, Marburg, Germany

**Keywords:** communication, death, dying, end-of-life, expectations, expectation violation, coping, ViolEx model

## Abstract

**Background:**

End-of-life (EOL) communication is often avoided, especially among young adults. Negative expectations concerning EOL conversations with relatives or significant others are one major reason.

**Objective:**

To investigate how best to violate negative expectations concerning EOL conversations by identifying predictors of coping with expectation violations in this context.

**Methods:**

Vignettes describing expectation violations in the context of EOL communication were presented to a sample of 261 university students. In a first experiment, the credibility of the expectation-disconfirming information was manipulated. In a second experiment, the valence of the disconfirming evidence was manipulated. As outcome measures, the subjective likelihood of two different responses to the expectation violation was assessed: (1) ignoring the disconfirming evidence (immunization) and (2) changing expectations (accommodation).

**Results:**

Overall, participants experiencing a worse-than-expected event showed more immunization [*F*(1, 257) = 12.15, *p* < 0.001, *η_p_* = 0.05], while participants experiencing a better-than-expected event showed more accommodation [*F*(1, 257) = 30.98, *p* < 0.001, *η_p_* = 0.11]. Participants with higher fear of death [*F*(1, 257) = 12.24, *p* < 0.001, *η_p_* = 0.05] as well as higher death avoidance tendencies [*F*(1, 257) = 17.16, *p* < 0.001, *η_p_* = 0.06] showed less accommodation in response to a better-than-expected event.

**Conclusion:**

In general, young adults appear to update their expectations quickly in response to unexpectedly positive experiences in the context of EOL communication. However, individuals with higher fear of death and higher death avoidance tendencies appear to be at higher risk of maintaining negative expectations despite disconfirming evidence.

## 1. Introduction

Numerous studies in the field of palliative care as well as in other fields have already demonstrated the high relevance of early onset end-of-life (EOL) communication for patients, relatives or even young and healthy individuals (Detering et al., 2010; Yamaguchi et al., 2017; Mroz et al., 2022). From the patient’s perspective, it leads to a higher congruence between EOL wishes and the care provided in their final stages of life (Detering et al., 2010; Mack et al., 2010). With regard to bereaved relatives, open EOL communication prior to the death of a loved one reduces the risk of developing mental health issues like anxieties, depression or complicated grief (Detering et al., 2010; Yamaguchi et al., 2017). Moreover, it is associated with various factors of personal growth after the death of a loved one, such as feeling more self-reliant with regard to handling the situation or strengthening relationships with other close persons (Generous and Keeley, 2022). Finally, early EOL communication is associated with less aggressive medical EOL care (Wright et al., 2008) resulting in both higher perceived quality of life in the terminal stages of a patient (Wright et al., 2008) as well as in lower costs for the health care system (Starr et al., 2019). It has further been demonstrated that even young and healthy individuals can benefit from dealing with the finiteness of their life. Becoming aware of one’s own mortality and engaging in EOL communication at an early stage of life can reduce death-related fears, increase insight into own EOL wishes, and lead to a clarification of personal values (Llewellyn et al., 2016; Mroz et al., 2022).

Despite the described benefits, EOL communication is often avoided or delayed until a person is no longer able to adequately express their wishes (Seifart et al., 2020). In an American representative survey, 92% of the respondents regarded having EOL conversations with their relatives as important (The Conversaton Project, 2018). However, only 32% of the surveyed had ever talked to relatives or significant others about their EOL wishes (The Conversaton Project, 2018). Studies with young adults show a similar pattern. In a survey among university students from the US, the majority of respondents reported positive attitudes towards EOL communication (Tripken and Elrod, 2018). However, only one third of the students had already engaged in EOL conversations (Tripken and Elrod, 2018). Thus, there seems to be a discrepancy between general attitudes towards EOL communication and reported behavior when it comes to actual conversations with loved ones.

There are various reasons for avoiding EOL topics in the family context, including emotional protection, relational characteristics as well as the deterioration of a person’s physical or mental condition (Generous and Keeley, 2017). Recent research in the field of EOL communication also suggests that negative expectations towards EOL conversations with a specific person might play an important role in this context (Bendel et al., 2022). On the one hand, most individuals expect a certain degree of emotional relief from EOL communication with a loved one (Von Blanckenburg et al., 2022). On the other hand, however, they expect being unable to discuss death-related topics in an adequate manner or to place high emotional strain on their conversation partner (Nagelschmidt et al., 2021; Von Blanckenburg et al., 2022). In line with these findings, 76% of US students in the aforementioned survey agreed with the statement that raising the topics of death and dying would make their conversation partner uncomfortable (Tripken and Elrod, 2018). The attempt to protect another person from negative emotions is often addressed in EOL communication literature, referred to as “protective buffering” (Nagelschmidt et al., 2021) or “emotional protection” (Generous and Keeley, 2017). It is considered to be a major avoidance factor with regard to EOL communication in the family context (Nagelschmidt et al., 2021). In summary, considering the role of expectations appears to be crucial in order to better understand the avoidance of EOL conversations with relatives or significant others.

Expectations are described in the literature as conditional beliefs about the probabilities of future events, experiences or information (Roese and Sherman, 2007; Hoorens, 2012). They can shape an individual’s behavior in anticipation of experiences or events in the future and are therefore considered to be a “highly relevant concept across basic and applied psychological disciplines” (Panitz et al., 2021). While engaging with their environment, individuals often have experiences or receive information that violate their original expectations. Effects of such expectation violations are the subject of scientific research and discussions, such as in communication research. While traditional views in this area tended to regard the effect of most expectation violations in communication as negative (Burgoon, 2015), Expectancy Violations Theory posits that positive expectation violations lead to desirable communication outcomes and thereby even outperform the effect of positive expectation confirmations (Burgoon, 2015).

With the ViolEx 2.0 model, Panitz et al. (2021) introduced a framework facilitating the investigation and understanding of expectation maintenance vs. change in the context of expectation violations. The model describes two possible ways of *coping* with expectation violations: *Accommodation* or *immunization*. While accommodation refers to “mechanisms by which individuals update their expectations following expectation violation,” immunization includes “mechanisms that aim at minimizing the impact of evidence disconfirming the original expectation and thereby prevent expectation update” (Panitz et al., 2021). While the term *coping* refers to dealing with a broad range of stressors and demands, such as dealing with the terminal diagnosis or death of a loved one (Generous and Keeley, 2021), the present study limits the focus to coping with a specific demand: violated expectations in the context of end-of-life communication.

A recent integrative review investigated a range of factors that predict coping with expectation violations (Pinquart et al., 2021). Among several other predictors, evidence has been found indicating that the credibility of the disconfirming information predicts coping with expectation violations. Evidence suggests that the higher the credibility of the disconfirming information, the more accommodation and expectation change occurs (Pinquart et al., 2021). In line with these findings, Kube et al. (2019b) showed that, when receiving additional information limiting the credibility of a test on “social competence,” participants did not change their performance expectations after receiving unexpectedly positive feedback in this test. However, when receiving no information about the test’s credibility, participants responded with accommodation and changed their prior expectations in a positive direction (Kube et al., 2019b).

The aforementioned review also found evidence suggesting that the valence of an expectation-disconfirming event affects coping with an expectation violation. According to Pinquart et al. (2021), evidence indicates that individuals are more likely to show accommodation and change their expectations when experiencing better-than-expected compared to worse-than-expected events. The reduced expectation update in response to worse-than-expected events might be explained by the occurrence of immunization processes (Kube et al., 2019a). Gesualdo and Pinquart (2022) investigated how the valence of an expectation-disconfirming event affects coping with expectation violations in the context of different health behaviors. In line with the findings of Pinquart et al. (2021) and Kube et al. (2019a), they could show that individuals experiencing a better-than-expected event related to physical activity responded with higher accommodation compared to individuals experiencing a worse-than-expected event. The latter, on the other hand, were more likely to respond with immunization. In summary, recent findings suggest that most individuals update their expectations and beliefs more quickly in response to better-than-expected evidence, while they try to shield them from worse-than-expected evidence.

Pinquart et al. (2021) also posit that personality characteristics of a person can influence how they cope with expectation violations. For example, there is some evidence showing that increased neuroticism and trait anxiety are associated with biased responses to expectation violations (Aue and Okon-Singer, 2015). Regarding the ViolEx 2.0 model, Pinquart et al. (2021) suggest that highly neurotic and anxious individuals generally tend to have more negative expectations about future events. If these events turn out to be even worse than expected, they are more likely to respond with accommodation. In contrast, highly anxious individuals are expected to immunize more strongly against better-than-expected experiences in order to maintain their preexisting negative expectations. In the field of EOL communication, two types of attitudes towards death and death-related events seem to be particularly relevant in light of these findings: First, the extent to which a person fears death; second, the extent to which they avoid death-related thoughts, feelings and situations (Jansen et al., 2019).

To summarize, EOL conversations are often avoided in the general population (The Conversaton Project, 2018). This applies above all to younger adults (Tripken and Elrod, 2018). Although most young adults subjectively perceive their own death as very distant in time, they are often confronted with death-related topics through aging relatives and are likely to become caregivers for them in the future (Tripken and Elrod, 2018). In order to better understand the avoidance of EOL conversations, negative expectations towards them should be considered and it should be investigated how these negative expectations can be violated (Bendel et al., 2022). However, there is little research investigating expectations towards EOL conversations using quantitative and experimental approaches (Von Blanckenburg et al., 2022). To the best of our knowledge, there is also no study explicitly addressing predictors of coping with expectation violations in this field.

Therefore, the aim of the present study was to identify factors predicting the coping with expectation violations in the context of EOL communication in a sample of university students. Following the approach of Gesualdo and Pinquart (2022), short vignettes describing expectation violations were presented to participants. According to Atzmüller and Steiner (2010), vignettes are a promising and effective tool to combine the benefits of traditional surveys (e.g., high external validity) and experimental designs (e.g., high internal validity). The vignettes used in the present study were developed with the support of a palliative care expert. They referred to an imagined conversation with a relative or other person important to the participating student. We chose this approach to ensure a high level of personal identification. In order to allow an expectation violation in both a positive and negative direction through the vignettes, participants were asked to select a person with whom they had not had much EOL communication experience before. After presentation of the vignettes, participants were asked to rate the subjective likelihood of two different reactions to the expectation violation described in the respective vignette (one representing accommodation and the other immunization). In a first experiment, we manipulated the credibility of the (positive) expectation-disconfirming information. In a second experiment, the valence of the expectation-disconfirming event was manipulated.

We hypothesized the following: While a lower credibility of expectation-disconfirming information will lead to higher immunization (hypothesis 1), a higher credibility will lead to higher accommodation (hypothesis 2). Moreover, we assumed that a worse-than-expected event will lead to higher immunization (hypothesis 3), while a better-than-expected event will lead to higher accommodation (hypothesis 4). Finally, we hypothesized that persons with (a) higher fear of death and (b) higher death avoidance tendencies will show more immunization if an event is better than expected (hypothesis 5a and 5b), while they will show more accommodation if an event is worse than expected (hypothesis 6a and 6b).

## 2. Materials and methods

### 2.1. Design and procedure

**Figure 1:**
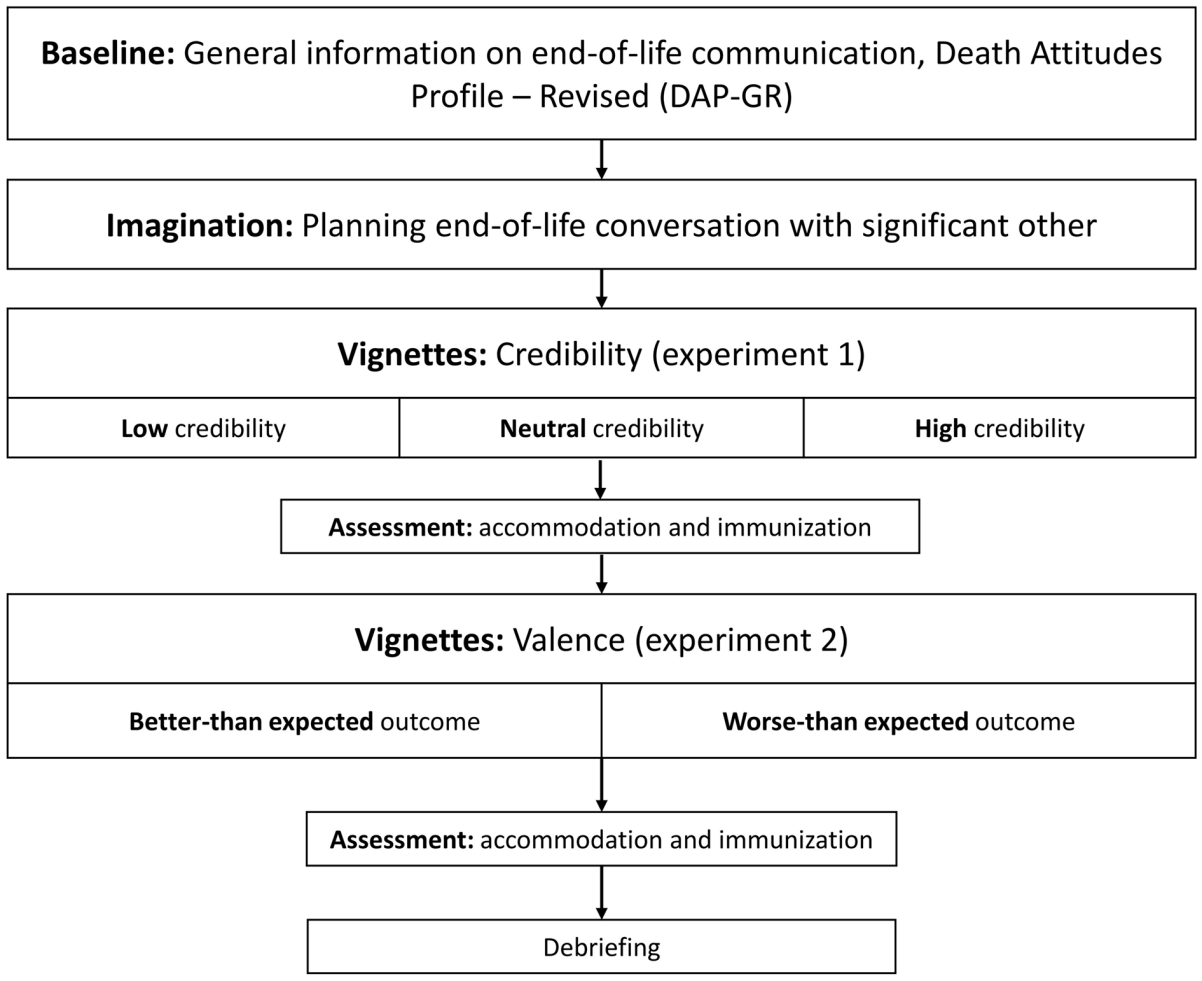
Procedure of the study.

The study was preregistered at the *Open Science Framework©* (Bendel and Gesualdo, 2022) and conducted online using *SoSci Survey©*. Recruitment took place in September and October 2022. University students with a minimum age of 18 years were primarily recruited *via* e-mail distribution lists of the researchers’ university. After providing informed consent, participants filled out a baseline questionnaire assessing sociodemographic characteristics as well as a questionnaire on their general attitudes towards death and dying. Moreover, they received general information about EOL communication to ensure a similar level of knowledge. Subsequently, participants were asked to imagine planning to have an EOL conversation with a significant other, in which the other person’s death will be addressed. In the first experiment, participants were then randomly assigned to one of three vignettes describing a positive, expectation-disconfirming experience in advance to an EOL conversation with the selected person. We intended to design these vignettes to represent three different levels of credibility (low, neutral or high). In the second experiment, participants were assigned to a vignette describing either a better-than-expected or worse-than-expected experience during an imagined EOL conversation with their selected person. All participants completed both experiments. In both experiments, accommodation as well as immunization were assessed in response to the vignette presented. The procedure is illustrated in Figure 1.

### 2.2. Content of the vignettes

#### 2.2.1. Vignettes in experiment 1

In the vignettes of the first experiment, participants were asked to imagine that they were conducting an internet search on EOL communication prior to the conversation with their selected person because they feared the emotional impact of the conversation. During this research, they came across the statement that EOL conversations are not too emotionally burdensome for most persons and, on the contrary, often even have an emotionally relieving effect. The credibility of this statement was manipulated by changing its author depending on the experimental condition, resulting in the presentation of three different vignettes (*low:* blog post by an unknown author, *neutral:* no information about the source and the author, *high:* statement of a palliative care expert). Appendix 1 presents the vignettes of experiment 1.

#### 2.2.2. Vignettes in experiment 2

In the vignettes of the second experiment, participants were asked to imagine that they were actually having a conversation with their selected person. The aim of this experiment was to manipulate the valence of an expectation-disconfirming event related to EOL communication. In a first vignette, participants were asked to imagine that they initially go into the conversation rather tense and nervous, but that it then turns out to be emotionally relieving (*better-than expected experience*). In a second vignette, they went into the conversation rather optimistically, but then became increasingly afraid of the other person’s death (*worse-than-expected experience*). Appendix 2 presents the vignettes of experiment 2.

### 2.3. Measures

#### 2.3.1. Sociodemographic characteristics and death attitudes

At the beginning of the study, participants completed a baseline questionnaire inquiring about age, gender, marital status and other sociodemographic variables. Attitudes towards death were assessed with the subscales *fear of death* and *death avoidance* of the German version of the Death Attitudes Profile – Revised (Jansen et al., 2019). The latter subscale includes items on the avoidance of death-related thoughts, feelings and situations. Items of both subscales were rated on a 7-point Likert scale ranging from 1 (“strongly disagree”) to 7 (“strongly agree”). Cronbach’s alpha was 0.89 for fear of death and 0.93 for death avoidance in this sample.

#### 2.3.2. Coping with expectation violations

Accommodation and immunization were assessed for each vignette using two self-developed items matching the respective vignette (e.g., accommodation: “I expect that future conversations about this topic will also be relieving for me.,” e.g., immunization: “This conversation may have been rather relieving. But that does not mean that every conversation will be like this.”). Items were developed based on the definitions of accommodation and immunization in the ViolEx 2.0 model (Panitz et al., 2021) and were formulated in line with other studies capturing these two coping strategies for dealing with expectation violations (Gesualdo and Pinquart, 2022). They were rated on a 6-point Likert scale ranging from 0 (“very unlikely”) to 5 (“very likely”).

#### 2.3.3. Validity of the vignettes

In order to evaluate the validity of the vignettes, participants were asked to indicate how well they could imagine the situation described in the respective vignette using a 6-point Likert scale ranging from 0 “not at all” to 5 “very well.”

### 2.4. Statistical analysis

Based on an *a priori* power analysis, we aimed to recruit a minimum of 207 participants in order to detect a medium effect size (*f* = 0.25, *α* = 0.05, power = 0.90). To test hypotheses 1 and 2, we used one-way ANOVAs (between-subjects factor: credibility). In order to test hypotheses 3, 4, 5a, 5b, 6a and 6b, we used 2 × 2 two-way ANOVAs. With a first two-way ANOVA, we investigated the interaction between valence and fear of death (between-subjects factor 1: valence, between-subjects factor 2: fear of death). With a second one, we investigated the interaction between valence and death avoidance (between-subjects factor 1: valence, between-subjects factor 2: death avoidance). To obtain the respective second dichotomous between-subjects factor, median splits were performed. In order to interpret effect sizes of main and interaction effects, *η_p_^2^* was calculated and inspected (small: *η_p_^2^* = 0.01, medium: *η_p_^2^* = 0.06, large: *η_p_^2^* = 0.14). All analyses were conducted using IBM SPSS Statistics Version 27.

## 3. Results

### 3.1. Sample characteristics

Data of *N* = 261 students were included in the analysis. The mean age was 23.03 years (*SD* = 4.42). Participants were predominantly female (72%) and German (89%). At the time of the survey, the majority of respondents had no partner (67%) and no children (98%). Approximately 69% already experienced the death of a close person and 62% reported having already engaged in an EOL conversation with another person about that person’s death. Sample characteristics are shown in Table 1.

**Table 1:** Sociodemographic characteristics of the participants (*N* = 261).

Variable	*N*	%
Mean age, years (SD)	23.03 (4.42)	–
Gender		
Female	188	72.03
Male	72	27.59
Non-binary	1	0.38
Nationality		
German	233	89.27
Other	28	10.73
Marital status		
Single	176	67.43
Married/partner	84	32.18
Divorced	1	0.38
Parenthood		
Yes	4	1.53
No	257	98.47
Death attitudes		
Mean (SD) / Mdn, fear of death	3.88 (1.44) / 4	–
Mean (SD) / Mdn, death avoidance	3.20 (1.41) / 3	–
Other		
Loss of a close person	180	68.97
Previous conversation about another person’s death	163	62.45

### 3.2. Experiment 1

#### 3.2.1. Validity of the vignettes

In experiment 1, participants reported that they could imagine the descripted situation “well” to “very well” (*M* = 4.44, *SD* = 0.93). No differences between the three experimental conditions were observed, *F*(2, 258) = 0.18, *p* = 0.837.

#### 3.2.2. Effect of credibility on immunization

The three groups (credibility low vs. neutral vs. high) did not differ in their level of immunization in response to the expectation-disconfirming experience, *F*(2, 258) = 0.25, *p* = 0.777. Means and standard errors by experimental condition are shown in Table 2.

**Table 2:** Accommodation and immunization by experimental condition, experiment 1 (*N* = 261).

Outcome measure	Credibility low	Credibility neutral	Credibility high
	*N* = 86, *M* (*SE*)	*N* = 86, *M* (*SE*)	*N* = 89, *M* (*SE*)
Immunization	2.44 (0.15)	2.37 (0.14)	2.52 (0.15)
Accommodation	3.21 (0.12)	3.29 (0.12)	3.26 (0.14)

M, mean; SE, standard error.

#### 3.2.3. Effect of credibility on accommodation

The three groups (credibility low vs. neutral vs. high) did not differ in their level of accommodation in response to the expectation-disconfirming experience, *F*(2, 258) = 0.10, *p* = 0.903. Means and standard errors by experimental condition are shown in Table 2.

### 3.3. Experiment 2

#### 3.3.1. Validity of the vignettes

In experiment 2, participants reported that they could imagine the descripted situation “well” to “very well” (*M* = 4.64, *SD* = 0.90). No differences between the two experimental conditions were observed, *F*(1, 259) = 1.02, *p* = 0.314.

#### 3.3.2. Effect of valence on immunization

Overall, participants experiencing a worse-than-expected event (*M* = 2.87, *SE* = 0.12) showed more immunization than participants experiencing a better-than-expected event (*M* = 2.27, *SE* = 0.13), *F*(1, 257) = 12.15, *p* < 0.001, *η_p_^2^* = 0.05.

#### 3.3.3. Effect of valence × fear of death on immunization

**Figure 2:**
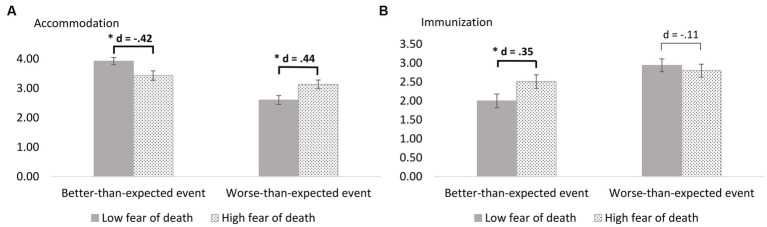
**(A)** Accommodation and **(B)** immunization after an expectation-violating event depending on valence of the event and fear of death, *N* = 261.

A trend towards an interaction of valence and fear of death on immunization was observed, *F*(1, 257) = 3.52, *p* = 0.062, *η_p_^2^* = 0.01. Compared to participants with lower fear of death, participants with higher fear of death tended to show more immunization in response to a better-than-expected event (Δ = 0.52, *p* = 0.049, *d* = 0.35). The results of valence and fear of death on immunization are illustrated in Figure 2. Means and standard errors by (quasi-)experimental condition are shown in Table 3.

**Table 3:** Accommodation and immunization by (quasi-)experimental condition, experiment 2: fear of death (*N* = 261).

Outcome measure	Better than expected, FoD low	Better than expected, FoD high	Worse than expected, FoD low	Worse than expected, FoD high
	*N* = 62, *M* (*SE*)	*N* = 68, *M* (*SE*)	*N* = 67, *M* (*SE*)	*N* = 64, *M* (*SE*)
Immunization	2.00 (0.18)	2.51 (0.18)	2.94 (0.17)	2.80 (0.17)
Accommodation	3.92 (0.12)	3.43 (0.16)	2.60 (0.15)	3.13 (0.15)

FoD, fear of death; M, mean; SE, standard error.

#### 3.3.4. Effect of valence on accommodation

Overall, participants experiencing a better-than-expected event (*M* = 3.66, *SE* = 0.10) showed more accommodation than participants experiencing a worse-than-expected event (*M* = 2.85, *SE* = 0.11), *F*(1, 257) = 30.98, *p* < 0.001, *η_p_^2^* = 0.11.

#### 3.3.5. Effect of valence × fear of death on accommodation

A significant interaction of valence and fear of death on accommodation was found, *F*(1, 257) = 12.24, *p* < 0.001, *η_p_^2^* = 0.05. Bonferroni-corrected post-hoc analysis revealed that, compared to participants with lower fear of death, participants with higher fear of death showed more accommodation in response to a worse-than-expected event (Δ = 0.53, *p* = 0.012, *d* = 0.44). In contrast, participants with higher fear of death showed less accommodation in response to a better-than-expected event (Δ = −0.49, *p* = 0.016, *d* = −0.42). The results of valence and fear of death on accommodation are illustrated in Figure 2. Means and standard errors by (quasi-)experimental condition are shown in Table 3.

#### 3.3.6. Effect of valence × death avoidance on immunization

**Figure 3:**
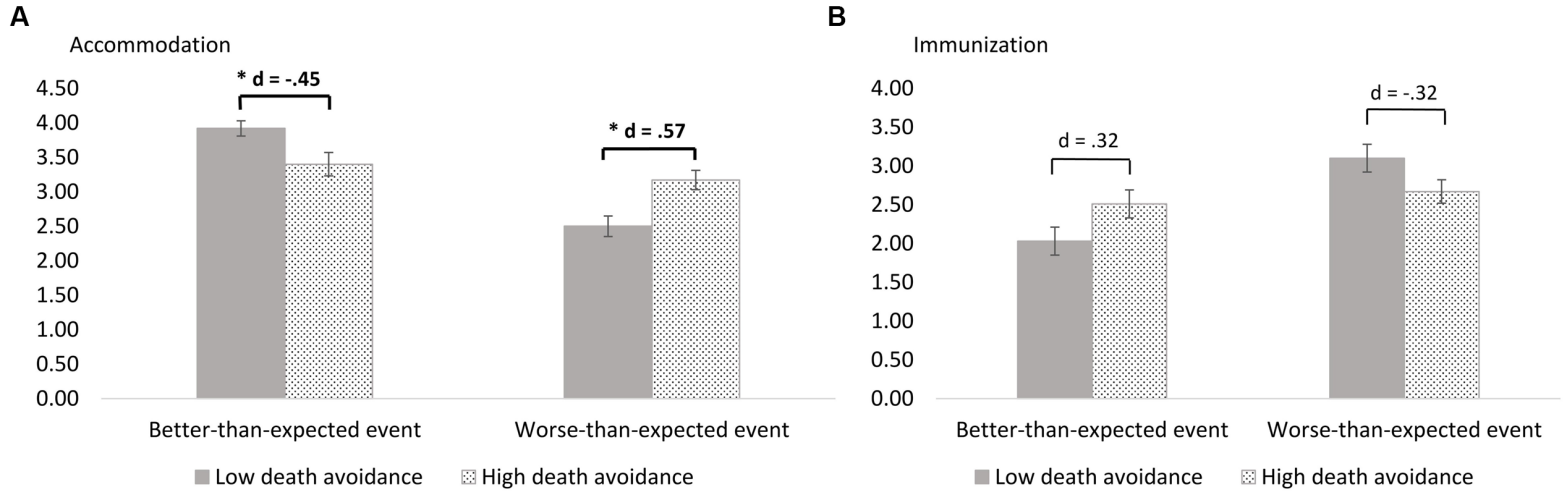
**(A)** Accommodation and **(B)** immunization after an expectation-violating event depending on valence of the event and death avoidance, *N* = 261.

A significant interaction of valence and death avoidance on immunization was observed, *F*(1, 257) = 6.74, *p* = 0.010, *η_p_^2^* = 0.03. Descriptively, participants with higher death avoidance tendencies showed more immunization in response to a better-than-expected event (*M* = 2.51, *SD* = 1.47) compared to participants with lower death avoidance tendencies (*M* = 2.03, *SD* = 1.49). However, in post-hoc analysis, mean differences did not reach significance (Δ = 0.48, *p* = 0.068, *d* = 0.32). The results of valence and death avoidance on immunization are illustrated in Figure 3. Means and standard errors by (quasi-)experimental condition are shown in Table 4.

**Table 4:** Accommodation and immunization by (quasi-)experimental condition, experiment 2: death avoidance (*N* = 261).

Outcome measure	Better than expected, DA low	Better than expected, DA high	Worse than expected, DA low	Worse than expected, DA high
	*N* = 65, *M* (*SE*)	*N* = 65, *M* (*SE*)	*N* = 62, *M* (*SE*)	*N* = 69, *M* (*SE*)
Immunization	2.03 (0.18)	2.51 (0.18)	3.10 (0.18)	2.67 (0.15)
Accommodation	3.92 (0.11)	3.40 (0.17)	2.50 (0.15)	3.17 (0.14)

DA, death avoidance; M, mean; SE, standard error.

#### 3.3.7. Effect of valence × death avoidance on accommodation

A significant interaction of valence and death avoidance on accommodation was observed, *F*(1, 257) = 17.16, *p* < 0.001, *η_p_^2^* = 0.06. Bonferroni-corrected post-hoc analysis revealed that, compared to participants with lower death avoidance tendencies, participants with higher death avoidance tendencies showed more accommodation in response to a worse-than-expected event (Δ = 0.67, *p* = 0.001, *d* = 0.57). In contrast, participants with higher death avoidance tendencies showed less accommodation in response to a better-than-expected event (Δ = −0.52, *p* = 0.011, *d* = −0.45). The results of valence and death avoidance on accommodation are illustrated in Figure 3. Means and standard errors by (quasi-)experimental condition are shown in Table 4.

## 4. Discussion

In this study, we investigated four predictors of coping with expectation violations related to (a) the disconfirming information or event (credibility and valence) and (b) characteristics of the person experiencing the expectation violation in the context of EOL communication (fear of death and death avoidance). In a first experiment, the credibility of the expectation-disconfirming information was manipulated. In a second experiment, we manipulated the valence of the expectation-disconfirming event.

With regard to experiment 1, we hypothesized that lower credibility would lead to more immunization, while higher credibility would lead to more accommodation. However, since we found no differences between the three experimental conditions, we were not able to confirm the first two hypotheses. This contradicts previous findings from other research areas, identifying the credibility of expectation-disconfirming information as an important predictor of coping with expectation violations (Kube et al., 2019b; Pinquart et al., 2021). The most probable explanation for this is that, in the present study, the experimental manipulation did not work as initially intended. The respective vignette described an expectation-violating statement the subject had read during an internet search on EOL communication with relatives (“However, once you get over yourself, you usually do not experience talking as excessively burdening, but rather as a relief”). We intended to manipulate credibility by changing the author of this statement depending on the experimental condition. While the statement in the “credibility low”-condition was a “blog post by an unknown author,” in the “credibility high”-condition it came from an “expert in the field of palliative care.” At this point, differences between experimental conditions might not have been sufficiently strong resulting in the absence of differences between groups in our outcomes. In order to emphasize the differences between conditions more strongly, the author in the “credibility low”-condition, for example, could have been presented in an even more untrustworthy manner (e.g., “The person who wrote this blog post has been accused in the past of spreading controversial theories and sometimes even lies”). However, another explanation for the lack of differences could be that previous results on credibility as a predictor of coping with expectations violations do not apply for EOL communication. The credibility of an externally received information might play a minor role in this context, as one’s own (imagined) experiences in a conversation and the emotions associated might be more important. In conclusion, more research is needed to further clarify the role of credibility with regard to expectation violations in EOL communication.

Hypotheses 3 and 4 were confirmed, since participants experiencing a worse-than-expected event in the context of EOL communication showed more immunization, while participants experiencing a better-than-expected event showed more accommodation. This is in line with previous findings indicating that individuals tend to update their expectations and beliefs more quickly in response to better-than-expected evidence, while they attempt to shield them from worse-than-expected evidence (Kube et al., 2019a; Gesualdo and Pinquart, 2022). The results also strengthen the claim of Expectancy Violations Theory that positive expectation violations, as opposed to negative ones, have a high potential to improve communication outcomes (Burgoon, 2015).

Finally, our results reinforce evidence for the influence of personality characteristics on coping with expectation violations (Pinquart et al., 2021). Participants with higher fear of death as well as higher death avoidance tendencies showed more immunization in response to a better-than-expected event in the context of EOL communication. In contrast, participants showed more accommodation in response to a worse-than-expected event. This corroborates the assumption of Pinquart et al. (2021) stating that anxious individuals immunize more strongly against unexpectedly positive experiences disconfirming their prior expectations, while they accommodate more strongly in response to worse-than-expected experiences.

### 4.1. Strengths and limitations

To the best of our knowledge, this study is the first explicitly addressing predictors of coping with expectation violations in the field of EOL communication research. Since, overall, there is limited research investigating expectations towards EOL communication using quantitative approaches (Von Blanckenburg et al., 2022), our study can make an important contribution in this area. The experimental approach, a sufficient statistical power as well as the online implementation ensured a high degree of standardization and allowed for reliable conclusions to be drawn. Moreover, this study examined young individuals, a social group that is often neglected in EOL communication research (Gerard, 2017). However, young individuals are often confronted with death-related topics through aging relatives and are likely to become caregivers for them in the future (Tripken and Elrod, 2018). Therefore, it is important to investigate how they deal with death-related topics and EOL conversations. Finally, our study investigated the influence of personal characteristics on coping with expectation violations in the EOL context. Understanding how different individuals respond to EOL issues is essential to overcoming conversational barriers and improving EOL communication between patients and caregivers through individualized approaches. Increasing early onset EOL communication could enable more patients to receive the EOL care they desire (Detering et al., 2010; Mack et al., 2010) and to experience a “good death” (Tenzek and Depner, 2017). This includes, but is not limited to, appropriate pain management, clear decision-making and preparation for death (Steinhauser et al., 2000). Moreover, EOL communication provides the opportunity for meaning making, for example, by strengthening the relationship between a patient and their loved ones (Generous and Keeley, 2014; Nickels et al., 2023). Finally, open EOL communication prior to the death of a loved one reduces the risk for bereaved relatives to develop mental health issues like anxieties, depression or complicated grief (Detering et al., 2010; Yamaguchi et al., 2017).

Nevertheless, relevant study limitations remain. First, the study only examined university students and three-quarters of the participants were female, which limits the generalizability of the results. Future studies should investigate more diverse samples with regard to age, gender and educational level. Second, the vignettes only described hypothetical scenarios for EOL conversations. Future studies should attempt to use more naturalistic settings. Third, we measured our outcomes, accommodation and immunization, with only one item each. At this point, using a scale including multiple items could have increased reliability of the measurement. However, this was difficult to implement, since the outcome measure was a highly specific response to a particular situation described in the respective vignette.

## 5. Conclusion

End-of-life (EOL) communication is often avoided due to negative expectations, especially among young adults. However, this age group is likely to be confronted with death-related topics in the future, for example through aging relatives and becoming caregivers. In order to prevent the avoidance of EOL conversations when necessary, further understanding on how best to violate negative expectations concerning this type of conversations is needed. Therefore, in the present study, different predictors of coping with expectation violations were investigated in the context of EOL communication. Results suggest that, in general, young adults tend to update their expectations quickly in response to unexpectedly positive experiences in this area. However, individuals with higher fear of death as well as higher death avoidance tendencies appear to be at higher risk of maintaining their negative expectations despite better-than-expected evidence. Therefore, interventions in the context of EOL communication should specifically address EOL fears as well as the avoidance of death-related thoughts, feelings and situations. This could facilitate expectation change and, for example, increase the effectiveness of public engagement initiatives aiming at increasing EOL communication. Future research should investigate predictors of coping with expectation violations in more naturalistic settings such as real life EOL conversations. Moreover, predictors of coping should be investigated in a sample of elderly persons and with regard to conversations about one’s own death.

## Data availability statement

The datasets presented in this study can be found in online repositories. The names of the repository/repositories and accession number (s) can be found at: http://doi.org/10.17605/OSF.IO/K3J7Y.

## Ethics statement

The studies involving humans were approved by local ethics committee of the Department of Psychology, Philipps University of Marburg. The studies were conducted in accordance with the local legislation and institutional requirements. The participants provided their written informed consent to participate in this study.

## Author contributions

YB: Conceptualization, Formal analysis, Investigation, Project administration, Writing – original draft, Methodology. CG: Conceptualization, Methodology, Writing – review & editing. MP: Conceptualization, Funding acquisition, Supervision, Writing – review & editing. PB: Conceptualization, Funding acquisition, Supervision, Writing – review & editing.
