# Supplementary material for: Better than expected? Predictors of coping with expectation violations in the communication about death and dying
Source: Front Psychol. 2023 Oct 30;14:1256202. doi: 10.3389/fpsyg.2023.1256202 (PMC10654619; doi:10.3389/fpsyg.2023.1256202)
Supplement: Supplementary file 1 [file Data_Sheet_1.PDF]

Appendix 1 – Vignettes of experiment 1

Credibility: low

In the following, you will be presented with different scenarios and asked questions about each. Please try to imagine these scenarios as best as you can. Take a few moments to do this and, afterwards, please indicate how likely you are to react in the ways described below.

You assume that talking about death can become emotionally burdensome for yourself or the other person. To prepare yourself a little, you first do a little Internet research.

During your research, you come across the following statement **from a person on an Internet blog. You do not know who exactly this person is (e.g. age, gender) and how he:she came to this statement.**

“Many people are concerned that such conversations will put too much emotional strain on their relatives or on themselves. However, once you get over yourself, you usually don’t experience talking as excessively burdening, but rather as a relief.”

1) I am more confident now and assume that the conversation with her:him will not be overly burdensome for either of us. (*Accommodation* – 6-point Likert scale)
2) I say to myself: The fact that many people feel this way does not mean that it will be the same for my conversation partner and me. (*Immunization* – 6-point Likert scale)

Credibility: neutral

In the following, you will be presented with different scenarios and asked questions about each. Please try to imagine these scenarios as best as you can. Take a few moments to do this and, afterwards, please indicate how likely you are to react in the ways described below.

You assume that talking about death can become emotionally burdensome for yourself or the other person. To prepare yourself a little, you first do a little Internet research.

During your research, you come across the following statement.

“Many people are concerned that such conversations will put too much emotional strain on their relatives or on themselves. However, once you get over yourself, you usually don’t experience talking as excessively burdening, but rather as a relief.”

1) I am more confident now and assume that the conversation with her:him will not be overly burdensome for either of us. (*Accommodation* – 6-point Likert scale)
2) I say to myself: The fact that many people feel this way does not mean that it will be the same for my conversation partner and me. (*Immunization* – 6-point Likert scale)

Credibility: high

In the following, you will be presented with different scenarios and asked questions about each. Please try to imagine these scenarios as best as you can. Take a few moments to do this and, afterwards, please indicate how likely you are to react in the ways described below.

You assume that talking about death can become emotionally burdensome for yourself or the other person. To prepare yourself a little, you first do a little Internet research.

During your research, you come across the following statement **from an expert in the field of palliative care who works with patients in their final stages of life and has conducted several studies on end-of-life communication.**

“Many people are concerned that such conversations will put too much emotional strain on their relatives or on themselves. However, once you get over yourself, you usually don’t experience talking as excessively burdening, but rather as a relief.”

1) I am more confident now and assume that the conversation with her:him will not be overly burdensome for either of us. (*Accommodation* – 6-point Likert scale)
2) I say to myself: The fact that many people feel this way doesn’t mean that it will be the same for my conversation partner and me. (*Immunization* – 6-point Likert scale)

Appendix 2 – Vignettes of experiment 2

Valence: Better-than-expected event

Now you actually want to talk to the person. The following describes a possible course of this conversation and possible reactions to it. Please read the text carefully and indicate how likely the following thoughts will occur to you (0 - very unlikely to 5 - very likely).

At first, you enter the conversation somewhat tense and nervous. It scares you to talk so concretely about the death and dying of the other person. However, after you have finally overcome this, the conversation goes surprisingly well. While you are talking, you notice that the tension is increasingly falling away from you, and after the conversation you feel relieved. The other person also experiences the conversation as relieving.

1) I expect that future conversations about this topic will also be relieving for me. (*Accommodation* – 6-point Likert scale)
2) I say to myself: This conversation may have been rather relieving. But that doesn't mean that every conversation will be like this. (*Immunization* – 6-point Likert scale)

Valence: Worse-than-expected event

Now you actually want to talk to the person. The following describes a possible course of this conversation and possible reactions to it. Please read the text carefully and indicate how likely the following thoughts will occur to you (0 - very unlikely to 5 - very likely).

At first, you enter the conversation rather optimistically, because you think it is important to start talking about this topic. During the conversation, however, you notice that you are becoming increasingly tense and nervous because you are now really becoming aware of what it would be like if the other person really dies. This scares you. Although the conversation doesn’t throw you completely "off track", you nevertheless feel anxious to a certain extent afterwards.

1) I expect that future conversations about this topic will also be burdensome for me. (*Accommodation* – 6-point Likert scale)
2) I say to myself: This conversation may have been rather burdensome. But that doesn't mean that every conversation will be like this. (*Immunization* – 6-point Likert scale)
